# Effect of Chlorogenic Acid on Melanogenesis of B16 Melanoma Cells

**DOI:** 10.3390/molecules190912940

**Published:** 2014-08-25

**Authors:** Hao-Rong Li, Maidina Habasi, Lian-Zhen Xie, Haji Akber Aisa

**Affiliations:** 1 The Key Laboratory of Plant Resources and Chemistry of Arid Zone, Xinjiang Technical Institute of Physics and Chemistry, Chinese Academy of Sciences, Urumqi 830011, China; E-Mails: bluehads@163.com (H.-R.L.); maidina27@hotmail.com (M.H.); xielz327@163.com (L.-Z.X.); 2 State Key Laboratory Basis of Xinjiang Indigenous Medicinal Plants Resource Utilization, Xinjiang Technical Institute of Physics and Chemistry, Chinese Academy of Sciences, Urumqi 830011, China; 3 University of Chinese Academy of Sciences, Beijing 1008611, China

**Keywords:** chlorogenic acid, melanogenesis, tyrosinase, cell lytic solution, browning reaction

## Abstract

Chlorogenic acid (CGA), the ester formed between caffeic acid and l-quinic acid, is a widespread phenolic compound. It is part of the human diet, found in foods such as coffee, apples, pears, *etc*. CGA is also was widely used in cosmetics, but the effects of CGA on melanogenesis are unknown. In this study, we analyzed the effects of CGA on cell proliferation, melanin content and tyrosinase of B16 murine melanoma cells. Additionally, the enzymatic reactions of CGA in B16 melanoma cells lytic solution were detected by UV spectrophotometry. Results showed CGA at 30 and 60 μM significantly suppresses cell proliferation. 8-MOP at 100 μM significantly promotes cell proliferation, but CGA can counter this. Incubated for 24 h, CGA (500 μM) improves melanogenesis while suppressing tyrosinase activity in B16 melanoma cells or 8-methoxypsoralen (8-MOP) co-incubated B16 melanoma cells. After 12 h, B16 melanoma cell treatment with CGA leads to an increase in melanin accumulation, however, after 48 h there is a decrease in melanin production which correlates broadly with a decrease in tyrosinase activity. CGA incubated with lytic solution 24 h turned brown at 37 °C. The formation of new products (with a maximum absorption at 295 nm) is associated with reduction of CGA (maximum absorption at 326 nm). Therefore, CGA has its two sidesroles in melanogenesis of B16 melanoma cells. CGA is a likely a substrate of melanin, but the metabolic product(s) of CGA may suppress melanogenesis in B16 melanoma cells by inhibiting tyrosinase activity.

## 1. Introduction

**Figure 1:**
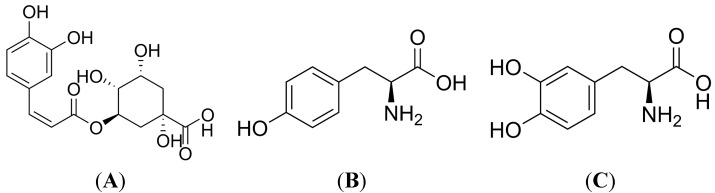
The chemical structures of chlorogenic acid, tyrosine and l-Dopa. (**A**) Chlorogenic acid; (**B**) Tyrosine; (**C**) l-Dopa.

Phenolic compounds are common in all fruits and vegetables. Chlorogenic acid (CGA) is the most important cinnamic acid derivative and accumulates to high levels in some crop plants, such as coffee, apples, pears, *etc.* CGA is also an important bioactive compound, abundant in some Traditional Chinese Medicines, such as flowers and buds of *Lonicera japonica*, thumb and the leaves of *Eucommia ulmoides*, *etc.* In 1932, Fischer and Dangschat deduced that CGA was 3-caffeoylquinic acid (3-CQA, Figure 1A). The biological activities of CGAs are numerous. CGA can exert anti-inflammatory effects. As an inhibitor of α-glucosidase, CGA is a promising candidate for the development of anti-type II diabetes and anti-AIDS drugs. The antioxidant activities of CGA are due to inhibition of the formation of reactive oxygen species (ROS) or by scavenging them. As a result, CGA is a polyphenol antioxidant that acts as a protective factor in oxidative stress-related diseases [1]. Decreasing ROS may prevent or minimize photocarcinogenesis and photoaging [2], so extracts rich of CGA are used in cosmetics [3,4]. Chlorogenic acid is oxidized by tyrosinase in the plant, which is known as polyphenol oxidase (PPO), to a highly reactive O-quinone intermediate which then can interact with the NH_2_ groups of lysines, SCH_3_ groups of methionines and the indole rings of tryptophanes in nucleophilic addition and in polymerization reactions, the so-called browning and greening reactions [5]. However, the effects of CGA on melanin synthesis are still unknown.

Melanocytes are present in the skin, hair and eyes, providing protection against ultraviolet (UV) light damage by absorbing UV sunlight and removing reactive oxygen species (ROS) [5]. Melanocyte loss or dysfunction results in depigmentation disorders and vitiligo [6]. On the other hand, increased activity of melanin-producing enzymes can lead to hyperpigmentation in humans and invertebrate animals [7]. The synthesis of melanin in melanocytes can be induced by many factors, including melanocyte stimulating hormone (α-MSH), cyclic adenosine monophosphate (cAMP)-elevating agents, UV light and 8-MOP [8,9,10]. UV light induces the tanning response, ROS and DNA damage [2,11]. UV light activates tyrosinase by microphthalmia-associated transcription factor (MITF) [12]. The most commonly clinically used psoralen is furanocoumarin that is widely used to treat vitiligo, psoriasis and other common skin diseases. 8-Methoxypsoralen (8-MOP) is the most potent inhibitor of cytochrome P-450 among furanocoumarins [13,14]. 8-MOP stimulates levels of microphthalmia-associated transcription factor (MITF) expression via PKA pathway, which in turn stimulates tyrosinase expression [15]. In order to avoid the ROS effect, we use 8-MOP to research melanogenesis in B16 murine melanoma cells.

Among tyrosinase (TYR), tyrosinase-related protein 1 (TRP-1) and tyrosinase-related protein 2 (TRP-2), tyrosinase plays the most critical role among melanogenic enzymes. TYR hydroxylates tyrosine (Figure 1B) into dihydroxyphenylalanine (DOPA, Figure 1C) and the further into DOPA quinone. DOPA chrome may be enzymatically transformed into 5,6-dihydroxyindole-2-carboxylic acid (DHICA) and 5,6-dihydroxyindole (DHI). DHI and DHICA are melanin precursors [16,17,18,19]. 

CGA is a substrate of PPO in browning reactions, but there is little data about the effect of CGA on melanocytes. This prompted us to investigate the potential of CGA to treat hypopigmentation (characterized by a loss of skin pigmentation) or hyperpigmentation (recognized by the presence of dark plaques on the skin).

## 2. Results and Discussion

### 2.1. Effects of CGA on the Proliferation of B16 Melanoma Cells

**Figure 2:**
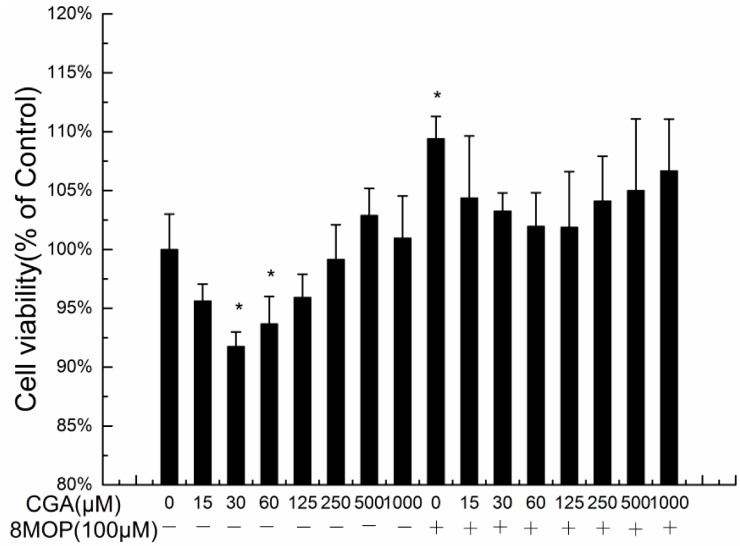
The effects of CGA on the cell proliferation of B16 melanoma cells and 8-MOP-treated B16 melanoma cells. Each value represents the mean ± SE (*n* = 3). * *p* < 0.05, compared with the control.

We evaluated the effects of CGA on the growth of B16 melanoma and 8-MOP-treated B16 melanoma cells. As seen in Figure 2, cell viability after treatment with CGA at 30 and 60 μM was significantly different from the control. The cell viability with 8-MOP at 100 μM was significantly different from the control. The cell viability with 8-MOP and CGA at 100 μM was significantly different from that of 8-MOP alone.

Consistent with the reported data, 8-MOP at 100 μM significantly promotes cell proliferation, but CGA can weaken this. The number of melanocytes in the epidermis can affect the skin pigmentation. The most lightly pigmented skin types (European, Chinese and Mexican) have approximately half as much epidermal melanin as the most darkly pigmented (African and Indian) skin types [20,21]. CGA at 30 and 60 µM concentration significantly suppressed cell proliferation, therefore, CGA inhibition of melanocyte proliferation may benefit in whitening the complexion.

### 2.2. Effects of CGA on Melanin Production and Tyrosinase Activity

**Figure 3:**
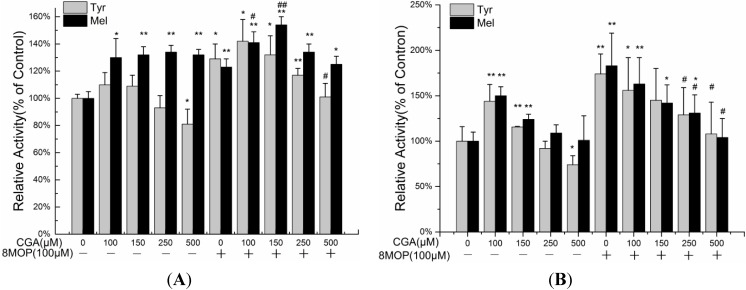
The effects of CGA on the melanin content (Mel) and tyrosinase activity (Tyr) of B16 melanoma cells and 8-MOP treated B16 melanoma cells, treated 24 h (**A**), 48 h (**B**). Each value represents the mean ± SE (*n* = 3). * *p* < 0.05, compared with the control. ** *p* < 0.01, compared with the control. # *p* < 0.05, compared with the 100 μM 8-MOP. ## *p* < 0.01, compared with the 100 μM 8-MOP.

Higher resistance to the effects of UV is a crucial aspect of cosmetics. The antioxidant activities of CGA have been reported. In order to avoid the antioxidant effect, we evaluated impacts of CGA on 8-MOP-treated B16 melanoma cells, in which tyrosinases were stimulated but do not produce H_2_O_2_. As reported CGA inhibited the formation of dopachrome from l-tyrosine or l-DOPA [22]. We hypothesized that CGA might inhibit tyrosinase activity. The results of this experiment are shown in Figure 3. Consistent with the literature [22], B16 melanoma cells incubated in 100 μM 8-MOP significantly stimulated melanogenesis compared with the control. After 24 h, the addition of CGA leads to an increase in melanin, which is not correlated with an increase in tyrosinase activity; therefore CGA is likely a product of some unidentified enzyme of the melanin biosynthetic pathway. At 500 µM CGA, tyrosinase activity was reduced significantly. Even with the addition of 8-MOP, CGA appears to reduce the activity of tyrosinase, while melanin accumulation remains high. At 48 h there appears to be a more obvious correlation between tyrosinase activity and melanin production in response to CGA treatments.

### 2.3. Effects of CGA on Cells Lysis Solution Enzymatic Reaction Assay

**Figure 4:**
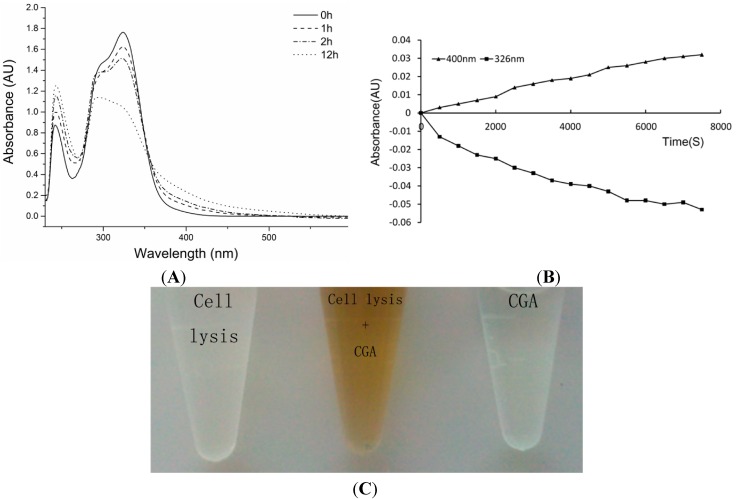
(**A**) Absorption spectra of CGA (0.5 mM) incubated with lytic solution (20 U/mL) at different incubation times; (**B**) Monitoring absorbance at 326 nm and 400 nm in time during incubations of CGA (0.5 mM) with lytic solution (20 U/mL); (**C**) CGA (0.5 mM) with lytic solution (20 U/mL) incubate 24 h in 37 °C.

Due to the fact the ectodomain of human tyrosinase expressed in *Escherichia coli* shows no influence towards chlorogenic acid [23], it is hypothesised that CGA might be substrate of melanogenic enzymes in B16 cells. It was not clear what kinds of enzyme(s) participate in the reaction, so CGA catalysis was initially researched using B16 melanoma cell lytic solution. As shown in Figure 4A, the absorption spectra of CGA incubated with cells lytic solution change over time.

After 2 h of incubation, a new absorption appeared at 295 nm, the absorption at 242 nm increased, and besides, the typical maximum absorption for CGA at 326 nm decreased. After 12 h of incubation, there is a maximum absorption at 295 and increases of the absorptions at 242 and 400 nm. In incubated CGA and cell lytic solution (Figure 4B) one can observe the formation of new products (absorption at 400 nm) with a decrease of CGA (absorption at 326 nm). The enzymatic oxidation of CGA by POD/H_2_O_2_ and PPO/O_2_ transforms the substrate into a new product with a maximum at 400 nm. Chlorogenic acid quinone (CQA-Q) may be responsible for the new absorption at 400 nm in the enzymatic reaction. CGA is inhibited by excess of substrate in enzymatic oxidation reactions [24,25]. CGA incubated with lytic solution 24 h turned brown at 37 °C (Figure 4C). l-Dopa incubated with lytic solution for 1 h turned brown. CGA is a competitive substrate in browning reactions.

## 3. Experimental Section

### 3.1. Materials

Chlorogenic acid, Triton X-100, mushroom tyrosinase, l-Dopa, dimethyl sulfoxide (DMSO), 3-(4,5-dimethylthiazol-2-yl)-2,5-diphenyltetrazolium bromide (MTT) and other chemicals were purchased from Sigma-Aldrich (St. Louis, MO, USA). Dulbecco’s modified Eagle’s medium (DMEM), fetal bovine serum (FBS) were purchased from Gibco (Grand Island, NY, USA). The deionized distilled water used in solutions and buffers was purified with a Milli-Q system (Millipore, Bedford, MA, USA).

### 3.2. Cell Culture and MTT Assay

B16 melanoma cells were purchased from the Type Culture Collection of the Chinese Academy of Sciences (Shanghai, China). The B16 melanoma cells were cultured in HG-DMEM, and then supplemented with 10% FBS, 100 mg/mL streptomycin and 100 U/mL penicillin. The cells were maintained in a humidified incubator with 5% CO_2_ at 37 °C, and they were sub-cultured every 2 days to maintain logarithmic growth. Cells were seeded in a 96-well plate at a density of 5 × 10^3^ cells/well. After 24 h of incubation, different concentrations of the test compounds were added to each well of the plate. After the plate was incubated for an additional 24 h, the attached cells were incubated with MTT (0.5 mg/mL, 1 h) and subsequently solubilized in DMSO. The absorbance at 550 nm was then measured using a micro-plate reader (SpectraMax M2Multi-Mode Microplate Reader, Molecular Devices, Sunnyvale, CA, USA) to calculate the percentage cell viability.

### 3.3. Tyrosinase Activity

B16 melanoma cells (5 × 10^4^ cells/well) were incubated in 24 well plates with various concentrations of the test compounds. After treatment with compounds, the cells were washed twice with phosphate buffered saline (PBS), and freeze-thaw lysed in 200 μL 0.1 M phosphate buffered saline (pH 6.8) containing 0.1%.Triton X-100. After protein quantification by BCA method, 100 μL of cell lytic solution (100 μg/mL) were mixed with 100 μL of 0.1% l-Dopa in phosphate buffer solution (pH 6.8), and incubated for 20 min at 37 °C. A spectrophotometric analysis was performed at 475 nm, using control cells as 100%, and the dopachrome formations of each sample were compared.

### 3.4. Melanin Content

B16 melanoma cells (5 × 10^5^ cells/dish) were incubated in 60 mm dishes with various concentrations of the test compounds. After treatment, the cells were washed twice with phosphate buffered saline (PBS), and lysed in 200 μL of 1 N NaOH for 1 h at 95 °C to solubilize the melanin. Lytic solution (100 μL) was added in a 96 well plate. The total amount of melanin was determined by enzyme micro-plate readings at 405 nm. The melanin content was calculated and corrected for the concentrations of proteins, using control cells as 100%.

### 3.5. Enzymatic Reaction

B16 melanoma cells were freeze-thaw lysed in 0.1 M phosphate buffered saline (pH 6.8) containing 0.1%.Triton X-100. Cell lysates mixed with CGA (0.5 mM) and incubated at 37 °C for 24 h. For spectrophotometric analysis, the same reaction mixtures were incubated in quartz cuvettes in a UV-visible spectrophotometer TU1901 (Purkinje, Beijing, China). Wavelength scans were made from 200 to 600 nm every 30 min.

### 3.6. General Procedures

General procedures were the same as in previous work [18,26,27,28] with some modifications. Assays were performed in triplicate on separate occasions as long as not specified otherwise.

### 3.7. Statistical Analysis

All analytical measurements were performed in triplicate. The results were analyzed using SPSS 19.0 and were expressed as the mean ± standard error for each measurement. *p*-values less than 0.05 were considered to be significant.

## 4. Conclusions

In summary, our experiments have demonstrated that CGA is involved in melanogenesis and affects the activity of tyrosinase. CGA can suppress cell proliferation accelerated by 8-MOP. CGA might be a substrate of melanogenic enzymes in B16 cells. CGA can suppress melanogenesis in B16 melanoma cells by inhibiting enzymatic oxidation of a diphenol, especially in B16 melanoma cells actived by 8-MOP. Tyrosine and l-DOPA are also bioregulatory agents acting not only as inducers and positive regulators of melanogenesis, but also as regulators of other cellular functions, so low concentrations of CGA can enhance melanogenesis and tyrosinase activity of B16 murine melanoma cells. With extendesd incubation time and increasing CGA concentration, the metabolic product(s) of CGA may suppress melanogenesis in B16 melanoma cells by inhibiting enzyme oxidation of a diphenol. CGA thus has its two roles in the melanogenesis of B16 melanoma cells.
